# Brain Metastases From Lung Adenocarcinoma May Preferentially Involve the Distal Middle Cerebral Artery Territory and Cerebellum

**DOI:** 10.3389/fonc.2020.01664

**Published:** 2020-08-28

**Authors:** Hyeokjin Kwon, Jun Won Kim, Mina Park, Jin Woo Kim, Minseo Kim, Sang Hyun Suh, Yoon Soo Chang, Sung Jun Ahn, Jong-Min Lee

**Affiliations:** ^1^Department of Biomedical Engineering, Hanyang University, Seoul, South Korea; ^2^Department of Radiation Oncology, Gangnam Severance Hospital, Yonsei University, College of Medicine, Seoul, South Korea; ^3^Department of Radiology, Gangnam Severance Hospital, Yonsei University, College of Medicine, Seoul, South Korea; ^4^Department of Internal Medicine, Gangnam Severance Hospital, Yonsei University, College of Medicine, Seoul, South Korea

**Keywords:** brain metastasis, lung cancer, adenocarcinoma, spatial distribution, arterial territory

## Abstract

Although whole-brain radiation therapy (WBRT) is the mainstay of treatment for brain metastases (BMs), the concept of saving eloquent cortical lesions has been promoted. If BMs from lung cancer are spatially biased to certain regions, this approach can be justified more. We evaluated whether BMs from lung cancer show a preference for certain brain regions and if their distribution pattern differs according to the histologic subtype of the primary lung cancer. In this retrospective study, 562 BMs in 80 patients were analyzed (107 BMs from small cell carcinoma, 432 from adenocarcinoma, and 23 from squamous cell carcinoma). Kernel density estimation was performed to investigate whether BM spatial patterns differed among lung cancer subtypes. Further, we explored more detailed subregions where BMs from adenocarcinomas occur frequently using one-way analysis of variance. Finally, we divided our cohort into those with fewer (≤10) and more (>10) BMs and evaluated whether this biased pattern was maintained across limited and extensive stages. For small cell carcinoma, BMs were biased to the cerebellum, but this did not reach statistical significance. For adenocarcinoma, BMs were found more frequently near the distal middle cerebral artery (MCA) territory and cerebellum than in other arterial territories (*p* < 0.01). The precentral and postcentral gyri were the most significant subregions within the distal anterior cerebral artery (ACA) and MCA territories (*p* < 0.01). Crus I and Lobule VI were significant regions within the cerebellum (*p* < 0.01). Regardless of the number of BMs, the affinity to the distal MCA territory and cerebellum was maintained. The present data confirm that BMs from lung adenocarcinoma may preferentially involve the distal MCA territory and cerebellum.

## Introduction

Brain metastases (BMs) are the most common intracranial neoplasms, outnumbering primary malignant brain tumors by more than 10-fold (1, 2). The incidence of BMs is still increasing because of the development of novel imaging techniques and improved survival rates (3). Lung cancer is the most frequent source of BMs, and 30–50% of patients with lung cancer develop BMs during the course of the disease (4). Although advanced therapies have improved the survival rates of lung cancer patients, BMs remain an important cause of morbidities associated with progressive neurologic deficits (5).

Whole-brain radiation therapy (WBRT) is currently the treatment of choice for patients with multiple BMs not amenable to surgery or radiosurgery. Although WBRT may prolong the survival of patients, long-term complications of WBRT could negatively impact their quality of life. Cognitive dysfunction is seen in 50–90% of patients who survive for more than 6 months after irradiation (6–8). Moreover, radiation-induced cognitive impairment occasionally progresses to dementia, where patients experience enough progressive memory loss to interfere with their daily lives (9). Hippocampal-sparing WBRT has been recently introduced to prevent radiation-induced cognitive deficits, which reduces the radiation dose to the hippocampus while applying the usual higher dose to other brain regions (10). In contrast, stereotactic radiosurgery (SRS) has been explored and is increasingly utilized for patients with limited BMs, as multiple Phase III randomized trials have demonstrated comparable overall survival and superior cognitive preservation and quality of life with SRS alone compared to SRS with WBRT (11–13). Further, the application of SRS has been expanded to patients with up to 10 BMs (14).

Interestingly, a few studies have reported the preferential involvement of BMs in certain regions. Early studies demonstrated the preferential involvement of BMs in anatomic watershed areas, the gray-white matter junction, and the cerebellum (15, 16). Tsukada et al. showed that breast cancer has a predilection for posterior fossa metastases compared with the cerebrum (17). Further, recent studies have suggested the possibility of different distribution patterns of BMs according to biological subtypes of primary cancer. BMs from lung cancer with epidermal growth factor receptor (EGFR) L858R mutations occurred more often in the caudate, cerebellum, and temporal lobe than those with an exon 19 deletion of *EGFR* (18). BMs from triple-negative breast cancers are evenly distributed in the brain; meanwhile, BMs from HER2-positive and luminal breast cancers occur primarily in the occipital lobe and cerebellum (19). If lung cancer BMs have different distribution patterns depending on histologic subtypes, radiation planning should be adjusted. Additionally, if lung cancer BMs show an affinity to certain brain regions, this knowledge could be applied to radiation treatment planning. Reducing the dose to the regions where BMs occur less frequently or low-dose WBRT with tumor boost may prevent the development of cognitive dysfunction, mood disorders, and motor impairment in patients (20).

However, previous studies for lung cancer BM distribution included patients with computed tomography (CT) or lower-field-strength (1.5 T) magnetic resonance imaging (MRI), which may miss small BMs (21). The sensitivity for the detection of BMs can be improved remarkably by increasing the field strength or gadolinium dose in MRI (22–24). They also performed lobar analysis, which might not reflect the mechanism of BMs and it might not be appropriate for finer radiation treatment planning.

In this study, we hypothesized that lung cancer BMs show a preferential involvement in certain brain regions and that their distribution pattern might differ according to histologic subtypes of primary lung cancer. To test this hypothesis, we generated probabilistic density functions of BMs and compared BM frequencies in arterial territories according to each histologic subtype. For adenocarcinoma, which is the most common subtype to metastasize to the brain (25) and which was associated with a significantly biased distribution of BMs in our analysis, we further investigated the regions where BMs occurred preferentially using a more detailed anatomical atlas.

## Materials and Methods

### Participants

This retrospective study was approved by our institutional review board, and the requirement to obtain informed patient consent was waived. We retrospectively reviewed data from 136 lung cancer patients with BMs who underwent gadolinium-enhanced brain MRI at our institution between October 2017 and December 2019. We excluded 56 patients for the following reasons: (1) previous neurosurgery or brain radiation therapy (*n* = 39), (2) presence of other malignant diseases (*n* = 8), (3) motion or dental material artifact on MR images (*n* = 4), and (4) a number of BMs per patient of more than 50 (*n* = 5) which was considered an outlier. All patients had histopathological diagnoses of lung cancer based on the results of bronchoscopic, percutaneous needle-guided, or surgical biopsies. After exclusion, 80 patients with 562 BMs remained. Recursive partitioning analysis (RPA) was used to classify patients into 1 of 3 categories: Class I included patients with Karnofky performance status (KPS) ≥70, age <65 years, controlled primary tumor, and no extracranial metastases; Class III included patients with a KPS <70; and Class II included all other patients (26). All data were fully anonymized, and all experiments were carried out in accordance with approved guidelines.

### MRI and Preprocessing

Routine images for the evaluation of BMs were acquired using a 3-T MR system (Vida, Siemens Healthineers; Erlangen, Germany) with a 64-channel head/neck-matrix coil. T1-weighted three-dimensional (3D) magnetization-prepared rapid acquisition with gradient echo (MPRAGE) images were obtained. After administering a 0.2 mmol/kg dose of gadobutrol (Gadovist, Bayer Schering Pharma; Berlin, Germany), 3D turbo spin-echo T1-weighted imaging (SPACE) was acquired. Sequence parameters for 3D T1-weighted MPRAGE were as follows: inversion time (TI) = 900 ms, echo time (TE) = 3 ms, repetition time (TR) = 2,300 ms, flip angle = 9°, slice thickness = 1 mm, FOV = 256 mm, matrix = 256 × 256, slice thickness = 1 mm, generalized autocalibrating partial parallel acquisition (GRAPPA) = 2, and time of acquisition = 5 min 12 s; for 3D T1-weighted SPACE: TE = 33 ms, TR = 700 ms, slice thickness = 0.8 mm, FOV = 230 mm, matrix = 288 × 288, slice thickness = 0.8 mm, acceleration factor of compressed sensing = 9, and time of acquisition = 3 min 44 s.

**FIGURE 1:**
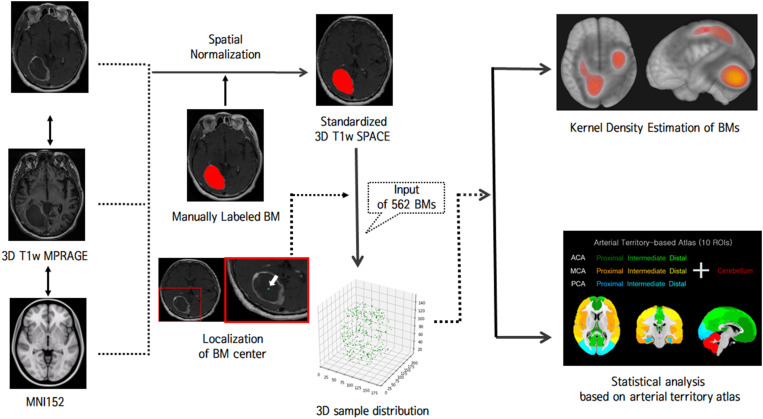
Schematic illustration of the flow of image analysis.

MR images were processed using the FMRIB Software Library^1^. For labeling BMs, 3D BM volumes were manually segmented on the native 3D T1-weighted SPACE images by a neuroradiologist. Binary labels of BMs were transformed into the Montreal Neurological Institute (MNI) space by the following process (Figure 1): 3D T1-weighted MPRAGE images were co-registered to gadolinium-enhanced 3D T1-weighted SPACE images using a rigid body transformation. The native 3D T1-weighted MPRAGE images were transformed to the standard MNI 152 T1-1-mm brain model using an affine transform matrix (27). The estimated transform matrix was concatenated with a co-registration transform matrix, and the resulting matrix was then applied to 3D T1-weighted SPACE images and binary BM label images. Finally, for each BM, voxels for the centers of gravity were localized, and all BMs were represented as single voxels in 3D space.

### Multivariate Non-parametric Kernel Density Estimation

Multivariate non-parametric kernel density estimation was performed to visualize the distribution of BM samples (28, 29). Multivariate non-parametric kernel density estimation enables better exploratory data analysis by reconstructing probabilistic density functions using the sample data in a non-parametric way (30, 31). A multivariate probabilistic density function $\hat{f}\,\left(x\right)$ is estimated as:

(1) $$\hat{f}\,\left(x;\text{Ω}\right)=\frac{1}{N}\,\sum_{i=1}^{N}{\text{K}}_{\text{Ω}}\,\left(x-X_{i}\right),{\text{K}}_{\text{Ω}}\,\left(x\right)={\left(2\,\pi \right)}^{-D/2}\,{\left|\text{Ω}\right|}^{-1/2}e^{-\frac{1}{2}\,x^{T}\,{\text{Ω}}^{-1}\,x}$$

where *X*_*i*_(*i* = 1,2,…,*N*; *N* is the number of samples) is a vector of size 1 × D containing multivariate sample and K^Ω^ is a kernel which we assumed to be a multivariate Gaussian variable with a diagonal bandwidth matrix Ω∈ℛ^*D*^. The Gaussian kernel is the most popular and is known for generating smooth and realistic densities (32, 33). The bandwidth matrix was estimated using a simple Silverman’s rule-of-thumb since we intended to visualize BM distributions, rather than perform quantitative analysis: (34)

(2) $$\omega_{d}={\left(\frac{4}{d+2}\right)}^{1/\left(d+4\right)}\,n^{-1/\left(d+4\right)}\,\sigma_{d}$$

where ω_*d*_ (*d* = 1, 2, …, *D*) is an estimated diagonal bandwidth and σ_*d*_ is the standard deviation of the data samples along the *d-th* dimension.

### Statistical Analysis

For each region of interest (ROI), a binary region mask of 5 mm was dilated to consider the origin of BMs more accurately and to standardize the chronological development of BMs (18). We then counted the number of BM samples in each region of the arterial-territory-based atlas to encode spatial patterns of BMs for each subject (35, 36). One-way analysis of variance (ANOVA) was performed to investigate whether the BM frequency differed among arterial territories according to each lung cancer subtype. Age, sex, and the area of each ROI were considered as covariates in the general linear models for ANOVA. To explore the more detailed anatomical hot spots of BMs from adenocarcinomas, which were found to be associated with a significantly biased pattern of BMs in a previous step, we applied the Harvard-Oxford probabilistic atlas to the distal MCA and ACA territories and the SUIT probabilistic cerebellum lobar atlas to the cerebellum (37, 38). Then, we compared BM frequencies between each subregion using one-way ANOVA. The probabilistic cerebellum lobar atlas was simplified by merging the left and right sides for each lobule, and both probabilistic atlases were thresholded at 25% prior to analyzing BM frequency. The significance level was set at *p* < 0.05 (39). To evaluate if biased patterns of BMs from adenocarcinoma were maintained across limited and extensive stages, we divided our cohort into those with a small number of BMs and those with a large number of BMs and compared BM frequencies of arterial territories in each group using one-way ANOVA.

## Results

### Different Spatial Distributions of Lung Cancer BMs in Different Histologic Subtypes

**FIGURE 2:**
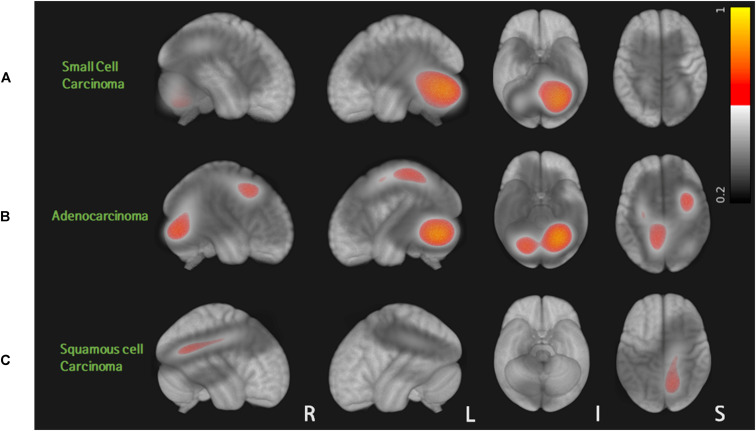
Probabilistic density function of brain metastases from three different histologic types of lung cancer: for small cell carcinoma, BMs were frequently found in the cerebellum **(A)**. For adenocarcinoma, BMs were clustered in the cerebellum, distal MCA, and ACA territories **(B)**. For squamous cell carcinoma, BMs were frequently observed in the right parietal lobe **(C)**.

A total of 80 patients with 562 metastases were included in this study: 13 cases of small cell carcinoma with 107 BMs, 58 cases of adenocarcinoma with 432 BMs, and 9 cases of squamous cell carcinoma with 23 BMs. Table 1 summarizes patient characteristics. Sex, smoking history, RPA results, and BM volume were significantly different among the three groups (*p* < 0.05). Probabilistic density functions showed different spatial distribution of BMs according to histologic subtypes of primary lung cancer (Figure 2). For small cell cancer, BMs were frequently found in the cerebellum. For adenocarcinoma, BMs were clustered in the cerebellum, distal MCA, and ACA territories. For squamous cell carcinoma, BMs were frequently observed in the right parietal lobe.

**TABLE 1:** Characteristics of lung cancer patients with brain metastases.

Characteristics	Small cell carcinoma (*N* = 13)	Adenocarcinoma (*N* = 58)	Squamous cell carcinoma (*N* = 9)	*p*-value
Age (years)	67.08 ± 9.32	65.78 ± 10.74	70.11 ± 8.46	0.61
**Sex**				0.05
Male	12 (92.3%)	21 (36.2%)	1 (11.1%)	
Female	1 (7.7%)	37 (63.8%)	8 (88.9%)	
**Smoking history**				0.01
Yes	9 (75%)	24 (43.6%)	8 (88.8%)	
No	3 (25%)	31 (56.4%)	1 (11.2%)	
**RPA**				0.01
1	0 (0%)	0 (0%)	1 (11.1%)	
2	8 (72.7%)	49 (90.7%)	5 (55.5%)	
3	3 (27.3%)	5 (9.3%)	3 (33.4%)	
**Number of BMs**	107	432	23	
Number of BMs per patient	8.23 ± 11.00	7.45 ± 7.41	2.56 ± 2.07	0.13
BM volume (mm^3^)	544.36 ± 2767.97	350.86 ± 1795.51	4097.30 ± 15793.24	0.04

*RPA, recursive partitioning analysis, BM, brain metastasis.*

### Different Frequencies of Lung Cancer BMs According to Arterial Territories in Different Histologic Subtypes

**FIGURE 3:**
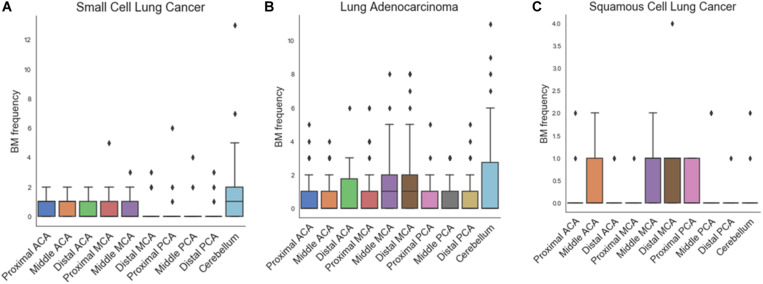
Box plots of frequency of brain metastases in arterial territories for small cell carcinoma **(A)**, adenocarcinoma **(B)**, and squamous cell carcinoma **(C)**.

Figure 3 and Table 2 show the results of one-way ANOVA for BM frequency among arterial territories. To better understand the spatial density of BMs, this model was adjusted for age, sex, and the areas of arterial territories. In adenocarcinoma, BMs were spatially biased (*p* < 0.01). According to post-hoc analysis, there were significantly more BMs in the distal MCA territory (1.79 ± 2.24) than in the proximal ACA (0.67 ± 1.14, *p* < 0.01), middle ACA (0.66 ± 1.04, *p* < 0.01), proximal MCA (0.86 ± 1.46, *p* = 0.04), proximal posterior cerebral artery (PCA) (0.60 ± 0.91, *p* < 0.01), middle PCA (0.71 ± 0.97, *p* < 0.01), or distal PCA territories (0.64 ± 1.09, *p* < 0.01, Supplementary Table 1). There were significantly more BMs in the cerebellum (1.62 ± 2.54) than in the proximal ACA (0.67 ± 1.14, *p* = 0.03), middle ACA (0.66 ± 1.04, *p* = 0.02), proximal PCA (0.60 ± 0.91, *p* = 0.01), or distal PCA territories (0.64 ± 1.09, *p* = 0.02). In small cell carcinoma, there was a tendency to have more BMs in the cerebellum compared with the other regions, but this was not significant (*p* = 0.07). There were no significant spatial biases of BMs in squamous cell carcinoma.

**TABLE 2:** Frequency of brain metastases according to histologic subtypes of the primary lung cancer by arterial territories.

Histology	ACA			MCA			PCA			CB	*p*-value
	P	M	D	P	M	D	P	M	D		
Small cell carcinoma	0.69 ± 0.72	0.62 ± 0.62	0.38 ± 0.62	0.77 ± 1.42	0.69 ± 0.91	0.54 ± 1.01	0.69 ± 1.64	0.46 ± 1.15	0.46 ± 0.93	2.46 ± 3.67	0.07
Adenocarcinoma	0.67 ± 1.14	0.66 ± 1.04	0.88 ± 1.23	0.86 ± 1.46	1.47 ± 1.76	1.79 ± 2.24	0.60 ± 0.91	0.71 ± 0.97	0.64 ± 1.09	1.62 ± 2.54	<0.01*
Squamous cell carcinoma	0.33 ± 0.67	0.67 ± 0.82	0.22 ± 0.42	0.11 ± 0.31	0.89 ± 0.74	1.00 ± 1.15	0.33 ± 0.47	0.44 ± 0.83	0.11 ± 0.31	0.33 ± 0.67	0.11

One-way analysis of variance was used to compare BM frequencies among arterial territories. This model was adjusted for age, sex, and the area of the arterial territory. Data shown are mean BM frequencies with standard deviations. Asterisk (*) indicates a *p*-value <0.05. ACA, anterior cerebral artery; BM, brain metastasis; MCA, middle cerebral artery; PCA, posterior cerebral artery; CB, cerebellum; P, proximal; M, middle; D, distal.

### Comparison of BM Frequency by Subregions of Distal ACA and Distal MCA Territories in Adenocarcinoma

**FIGURE 4:**
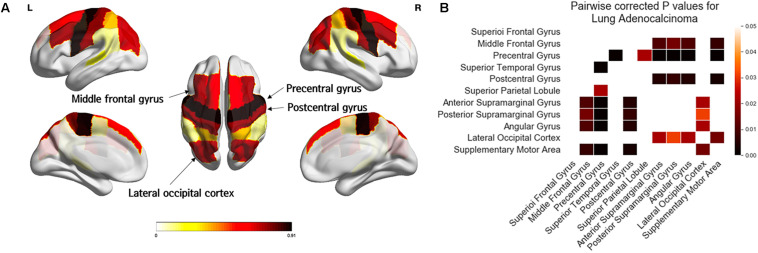
Comparison of the frequency of brain metastases from adenocarcinoma by subregions of the distal ACA and MCA territories. Yellow regions indicate a low frequency of brain metastases (BMs) while black regions indicate a high BM frequency **(A)**. Tukey’s HSD post-hoc pairwise test for comparison of BM frequency between subregions in the distal ACA and MCA territories. All colored cells indicate a significant difference between two regions (*p* < 0.05). Darker colors indicate greater differences in BMs between two regions **(B)**. ACA; anterior cerebral artery; MCA, middle cerebral artery.

Figure 4 and Table 3 showed the results of one-way ANOVA for BM frequency among subregions of the distal MCA and ACA territories. The covariates were age, sex, and areas of the subregions. In the distal ACA and MCA territories, BMs were significantly biased in certain subregions (*p* < 0.01). BMs were found most frequently in the precentral gyrus, followed by the postcentral gyrus, middle frontal gyrus, and lateral occipital cortex. According to post-hoc analysis, there were significantly more BMs in the precentral gyrus (0.91 ± 1.51) than in the superior temporal gyrus (0.22 ± 0.74, *p* < 0.01), superior parietal lobule (0.34 ± 0.66, *p* = 0.02), anterior supramarginal gyrus (0.10 ± 0.36, *p* < 0.01), posterior supramarginal gyrus (0.12 ± 0.46, *p* < 0.01), angular gyrus (0.10 ± 0.36, *p* < 0.01), or supplementary motor area (0.09 ± 0.28, *p* < 0.01, Supplementary Table 2). There were significantly more BMs in the postcentral gyrus (0.74 ± 1.14), the middle frontal gyrus (0.71 ± 1.22), and the lateral occipital cortex (0.67 ± 0.94) than in the anterior supramarginal gyrus (0.10 ± 0.36), posterior supramarginal gyrus (0.12 ± 0.46), angular gyrus (0.10 ± 0.36), and supplementary motor area (0.09 ± 0.28, *p* < 0.05).

**TABLE 3:** Frequency of brain metastases from adenocarcinoma by subregions of the distal ACA and MCA territories.

Distal ACA, MCA	SFG	MFG	PreG	STG	PostG	SPL	ASG	PSG	AG	LOC	SMA	*p*-value
	0.57 ± 0.93	0.71 ± 1.22	0.91 ± 1.51	0.22 ± 0.74	0.74 ± 1.14	0.34 ± 0.66	0.10 ± 0.36	0.12 ± 0.46	0.10 ± 0.36	0.67 ± 0.94	0.09 ± 0.28	<0.01

*This model was adjusted for age, sex, and the area of the arterial territory. Data shown are mean frequencies of brain metastases with standard deviations. ACA, anterior cerebral artery; MCA, middle cerebral artery; SFG, superior frontal gyrus; MFG, middle frontal gyrus; PreG, precentral gyrus; STG, superior temporal gyrus; PostG, postcentral gyrus; SPL, superior parietal lobule; ASG, anterior supramarginal gyrus; PSG, posterior supramarginal gyrus; AG, angular gyrus; LOC, lateral occipital cortex; SMA, supplementary motor area.*

### Comparison of BM Frequency by Subregions of the Cerebellum in Adenocarcinoma

**FIGURE 5:**
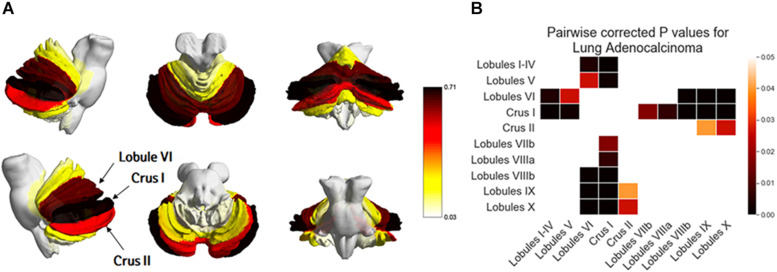
Comparison of the frequency of brain metastases from adenocarcinoma by subregions of the cerebellum. Yellow regions indicate a low frequency of brain metastases (BMs) while black regions indicate a high BM frequency **(A)**. Tukey’s HSD post-hoc pairwise test for the comparison of BM frequency between subregions of the cerebellum. All colored cells indicate a significant difference between two regions (*p* < 0.05). Darker colors indicate a greater difference in BMs between two regions **(B)**.

One-way ANOVA showed that BMs were significantly biased to certain subregions of the cerebellum (*p* < 0.01, Figure 5 and Table 4). BMs were found most frequently in Crus I, followed by Lobule VI and Crus II. According to post-hoc testing, there were significantly more BMs in Crus I (0.71 ± 1.16) than in Lobules I-IV (0.10 ± 0.44, *p* < 0.01), V (0.17 ± 0.53, *p* < 0.01), VIIb (0.24 ± 0.43, *p* = 0.01), VIIIa (0.21 ± 0.41, *p* < 0.01), VIIIIb (0.07 ± 0.25, *p* < 0.01), IX (0.05 ± 0.29, *p* < 0.01), or X (0.03 ± 0.26, *p* < 0.01, Supplementary Table 3). There were significantly more BMs in Lobule VI (0.62 ± 1.35) than in Lobules I-IV (0.10 ± 0.44, *p* < 0.01), V (0.17 ± 0.53, *p* = 0.02), VIIIIb (0.07 ± 0.25, *p* < 0.01), IX (0.05 ± 0.29, *p* < 0.01), or X (0.03 ± 0.26, *p* < 0.01). There were significantly more BMs in Crus II (0.48 ± 0.90) than in Lobule IX (0.05 ± 0.29, *p* = 0.03) or X (0.03 ± 0.26, *p* = 0.02).

**TABLE 4:** Frequencies of brain metastases from adenocarcinoma by subregions of the cerebellum.

CB	LB I-IV	LB V	LB VI	Crus I	Crus II	LB VIIb	LB VIIIa	LB VIIIb	LB IX	LB X	*p*-value
	0.10 ± 0.44	0.17 ± 0.53	0.62 ± 1.35	0.71 ± 1.16	0.48 ± 0.90	0.24 ± 0.43	0.21 ± 0.41	0.07 ± 0.25	0.05 ± 0.29	0.03 ± 0.26	<0.01

*This model was adjusted for age, sex, and the area of the arterial territory. Data shown are mean frequencies of brain metastases with standard deviations. CB, cerebellum; LB, lobule.*

### BM Distribution in Patients With Fewer BMs (≤10) and Those With More BMs (>10) From Adenocarcinoma

In 57 patients with 1–10 BMs from adenocarcinoma, BMs were found most frequently in the cerebellum followed by the distal MCA and middle MCA territories (Table 5). There were significantly more BMs in these arterial territories (cerebellum: 0.74 ± 1.28, distal MCA: 0.72 ± 1.06, middle MCA: 0.54 ± 0.63) than in other territories (*p* = 0.02, Supplementary Table 5). In 23 patients with more than 10 BMs from adenocarcinoma, BMs were found most frequently in the distal MCA territory, followed by the cerebellum and middle MCA territory. There were significantly more BMs in these arterial territories (distal MCA: 4.0 ± 2.41, cerebellum: 3.42 ± 3.41, middle MCA: 3.37 ± 1.81) than in other territories (*p* < 0.01, Supplementary Table 6).

**TABLE 5:** Arterial distribution patterns in patients with fewer BMs (≤10) and those with more BMs (>10).

Number of BMs	ACA			MCA			PCA			CB	*p*-value
	P	M	D	P	M	D	P	M	D		
≤10	0.38 ± 0.84	0.28 ± 0.6	0.33 ± 0.61	0.28 ± 0.6	0.54 ± 0.63	0.72 ± 1.06	0.23 ± 0.42	0.26 ± 0.49	0.28 ± 0.64	0.74 ± 1.28	0.02*
>10	1.26 ± 1.41	1.42 ± 1.31	2.0 ± 1.41	2.05 ± 1.9	3.37 ± 1.81	4.0 ± 2.41	1.37 ± 1.13	1.63 ± 1.04	1.37 ± 1.42	3.42 ± 3.41	< 0.01*

*One-way analysis of variance was used to compare BM frequencies among arterial territories. This model was adjusted for age, sex, and the area of the arterial territory. Data shown are mean frequencies of brain metastases with standard deviations. Asterisk (^∗^) indicates a *p*-value <0.05. BM, brain metastasis; ACA, anterior cerebral artery; MCA, middle cerebral artery; PCA, posterior cerebral artery; CB, cerebellum; P, proximal; M, middle; D, distal.*

## Discussion

In this study, we tested the hypothesis that lung cancer BMs may be spatially biased to certain brain regions and that their distribution patterns differ between the lung cancer subtypes of small cell carcinoma, adenocarcinoma, and squamous cell carcinoma. Our results indicated that BMs of certain subtypes may have different preferential areas of involvement in the brain. Specifically, in adenocarcinoma, BMs were more frequently found in the cerebellum and distal MCA territory than in other arterial territories, and this tendency was maintained from the limited to extensive stages of BMs. Furthermore, in the distal ACA and MCA territories, the precentral gyrus, postcentral gyrus, middle frontal gyrus, and lateral occipital cortex were hot spots for BMs. In the cerebellum, Crus I, Lobule VI, and Crus II were preferential subregions for BMs. Our study findings may shed light on the mechanism by which lung cancer spreads to the brain and may be utilized for tailoring radiation doses according to lung cancer histology.

The preferential involvement of BMs in the cerebellum has been reported in the previous literature. A previous CT-based study demonstrated that the posterior fossa was involved in 50% of patients when the primary tumor was pelvic or gastrointestinal, but it was involved in only 10% of patients with other primary tumors (15). However, recent MR-based studies showed an increased probability of cerebellar metastases in lung and breast cancer (40, 41). Our result also indicated that the cerebellum is the preferential site for BMs of small cell carcinoma and adenocarcinoma. The preferential involvement of the cerebellum has been explained by the retrograde pathway of BMs. The Batson venous plexus is a network of valveless veins that connect the deep pelvic and thoracic veins to internal vertebral venous plexuses. The cerebral dural sinuses are a direct extension of the spinal epidural plexus. Thus, the pathway via the Batson venous plexus to the cerebral dural sinuses can be an important route for BMs (42). This mechanism may provide an explanation for the occurrence of breast cancer and pelvic organ tumors, which communicate with the Batson plexus. However, it may not be applicable in lung cancer because lung cancer drains through the pulmonary vein to the heart and spreads to distant sites through direct arterial dissemination. Thus, the mechanism for the preferential involvement of the cerebellum in small cell carcinoma and adenocarcinoma is uncertain and further study is necessary.

Interestingly, our results demonstrated that histologic subtypes of lung cancer exhibit distinct distribution patterns of BMs. BMs of squamous cell carcinoma did not show a preferential involvement of the cerebellum. For small cell carcinoma, BMs were frequently observed in the cerebellum. For adenocarcinoma, BMs were frequently found not only in the cerebellum but also in the distal MCA territory. The differential distribution of BMs according to lung cancer subtype has been rarely studied. One study compared the frequency of BMs by histologic subtypes and *EGFR* mutation status of lung cancers. They found heterogeneity of BMs in small cell carcinoma and adenocarcinoma, but not in squamous cell carcinoma, which is in line with our study (18). It has been revealed that small cell carcinoma and adenocarcinoma tend to metastasize into the brain, whereas squamous cell carcinoma prefers bones (43). Although the mechanism for the preferential involvement of specific brain regions remains unknown, we speculate That “seed and soil theory” may partially explain it. The affinity of the tumor to the microenvironment plays a role in extravasation and colonization of tumor cells at specific sites (44). Recent studies support this notion at the molecular level. Matrix metalloprotease-2 (MMP-2) and MMP-9 have been reported to induce tumor cell invasion and metastasis formation (45). Plasmin acts as a defense against metastatic invasion. Neuroserpin and serpine B2 have been reported to prevent plasmin generation (46). Also chemokine receptors such as CXCR4 and CCR7 seem to play a critical role in determining the metastatic destination (47). The expression of these molecules may different depending on the subtypes of lung cancer, but this hypothesis should be verified in the future studies.

Our findings indicated that more BMs were found in the distal arterial territories (ACA and MCA territories) than in the proximal arterial territories. This finding supports the arterial dissemination of BMs, in agreement with the rule that tumoral emboli tend to pass along the arterial tree as far distally as their size permits (48). However, the reason for the greater affinity of BMs to the MCA territory than other arterial territories is not known. We presume that it might be associated with the higher blood flow rate of the MCA (21% of total cerebral flow rate) compared with other territories (ACA: 12% of total cerebral flow rate, PCA: 8% of total cerebral blood flow) (49).

Although WBRT was the mainstay of treatment for BMs, its role has diminished over the last several decades. SRS is preferred over WBRT for limited BMs because its efficacy is non-inferior with greater preservation of neurocognitive functioning and fewer serious adverse effects (13, 14). Even when WBRT is applied, anatomic avoidance strategies are promoted for cognitive preservation. Accumulating evidence has suggested that hippocampal-sparing WBRT reduces neurocognitive impairment (50, 51). Preserving the eloquent cortex is even more justified when it is used for prophylactic cranial irradiation. In this perspective, our results might be applied in planning WBRT to selectively spare structures where BMs occur less frequently. The precentral and postcentral gyri are significantly associated with motor and sensory functions which might severely affect patients’ quality of life. However, our subregion analysis demonstrated that the precentral and postcentral gyri were hot spots for BMs of adenocarcinoma within the distal MCA and distal ACA territories. Thus, a usual higher dose should be maintained in these regions. Furthermore, it is notable that certain cerebellar subregions (Lobule IV and Crus II) have more BMs than other regions. These regions are commonly supplied by the distal superior cerebellar artery (SCA). This may be due to a preference of BMs for the SCA compared to the anterior inferior cerebellar artery or the posterior cerebellar artery (52). Previous studies have revealed that cerebellar lesions can cause motor deficits as well as cognitive dysfunction. The anterior lobe is associated with motor skills whereas the posterior lobe is associated with cognition (53, 54). Thus, radiation therapy based on detailed information of BM distributions in the cerebellum may preserve motor or cognitive function.

In our study, the BM distribution pattern for adenocarcinoma did not differ between those with a small number of BMs and those with a large number of BMs, which indicates that whatever the mechanism of BM is, whether by arterial tumor emboli or a retrograde pathway, the concentration in the distal MCA territory and cerebellum is maintained from early to advanced stages of BMs. Thus, our results may support the use of localized radiation boosts to these regions.

This study had a limitation. Although there was a tendency that BMs from small cell carcinoma were found more frequently in the cerebellum than in other arterial territories, it was not significant. We presume that this is probably due to the relatively fewer number of BMs from small cell carcinoma than that of BMs from adenocarcinoma. Thus, future studies including more BMs are necessary to validate this tendency.

## Conclusion

In conclusion, our study demonstrated that BMs in lung adenocarcinoma were biased to the distal MCA territory and cerebellum. Within the distal ACA and MCA territories, the precentral gyrus, postcentral gyrus, middle frontal gyrus, and lateral occipital cortex were preferred subregions for BMs. Within the cerebellum, Crus I, Lobule VI, and Crus II were hot spots. Our results may not only enlighten the research on the mechanisms underlying metastatic dissemination in the brain but can also be used as a fundamental basis for developing tailored radiation therapies for BMs.

## Data Availability Statement

All datasets generated for this study are included in the article/Supplementary Material.

## Ethics Statement

The studies involving human participants were reviewed and approved by institution review board of Gangnam Severance Hospital. Written informed consent for participation was not required for this study in accordance with the national legislation and the institutional requirements.

## Author Contributions

SA and J-ML: study conception and design. JuK and YC: material preparation and data collection. HK and MK: data analysis. JiK, MP, and SS: result interpretation. All authors have significantly contributed to the manuscript, wrote and revised the manuscript.

## Conflict of Interest

The authors declare that the research was conducted in the absence of any commercial or financial relationships that could be construed as a potential conflict of interest.

## Footnotes

1 http://www.fmrib.ox.ac.uk/fsl
