# Corrigendum: Brain Metastases From Lung Adenocarcinoma May Preferentially Involve the Distal Middle Cerebral Artery Territory and Cerebellum

**DOI:** 10.3389/fonc.2020.610495

**Published:** 2020-11-06

**Authors:** Hyeokjin Kwon, Jun Won Kim, Mina Park, Jin Woo Kim, Minseo Kim, Sang Hyun Suh, Yoon Soo Chang, Sung Jun Ahn, Jong-Min Lee

**Affiliations:** ^1^ Department of Biomedical Engineering, Hanyang University, Seoul, South Korea; ^2^ Department of Radiation Oncology, Gangnam Severance Hospital, Yonsei University, College of Medicine, Seoul, South Korea; ^3^ Department of Radiology, Gangnam Severance Hospital, Yonsei University, College of Medicine, Seoul, South Korea; ^4^ Department of Internal Medicine, Gangnam Severance Hospital, Yonsei University, College of Medicine, Seoul, South Korea

**Keywords:** brain metastasis, lung cancer, adenocarcinoma, spatial distribution, arterial territory

In the published article, there was an error regarding the affiliation for Minseo Kim and Jong-Min Lee. Instead of affiliations 3 and 2, respectively, Minseo Kim and Jong-Min Lee should both have affiliation 1.

The authors apologize for these errors and state that this does not change the scientific conclusions of the article in any way. The original article has been updated.

