# Supplementary material for: Brain Metastases From Lung Adenocarcinoma May Preferentially Involve the Distal Middle Cerebral Artery Territory and Cerebellum
Source: Front Oncol. 2020 Aug 28;10:1664. doi: 10.3389/fonc.2020.01664 (PMC7484698; doi:10.3389/fonc.2020.01664)
Supplement: Supplementary file 1 [file Data_Sheet_1.docx]

Supplemental Table 1. Post-hoc analysis of one-way ANOVA for the comparison of frequencies of brain metastases from adenocarcinoma according to arterial territories

|  |  | ACA | | | MCA | | | PCA | | | CB |
| --- | --- | --- | --- | --- | --- | --- | --- | --- | --- | --- | --- |
|  |  | P | M | D | P | M | D | P | M | D |  |
| ACA | P | 1 | 0.9 | 0.9 | 0.9 | 0.15 | <0.01* | 0.9 | 0.9 | 0.9 | 0.03* |
|  | M | 0.9 | 1 | 0.9 | 0.9 | 0.13 | <0.01* | 0.9 | 0.9 | 0.9 | 0.02* |
|  | D | 0.9 | 0.9 | 1 | 0.9 | 0.56 | 0.04* | 0.9 | 0.9 | 0.9 | 0.23 |
| MCA | P | 0.9 | 0.9 | 0.9 | 1 | 0.52 | 0.04* | 0.9 | 0.9 | 0.9 | 0.20 |
|  | M | 0.15 | 0.13 | 0.56 | 0.52 | 1 | 0.9 | 0.08 | 0.20 | 0.11 | 0.9 |
|  | D | <0.01* | <0.01* | 0.04* | 0.04* | 0.9 | 1 | <0.01* | <0.01* | <0.01* | 0.9 |
| PCA | P | 0.9 | 0.9 | 0.9 | 0.9 | 0.08 | <0.01* | 1 | 0.9 | 0.9 | 0.01* |
|  | M | 0.9 | 0.9 | 0.9 | 0.9 | 0.20 | <0.01* | 0.9 | 1 | 0.9 | 0.04* |
|  | D | 0.9 | 0.9 | 0.9 | 0.9 | 0.11 | <0.01* | 0.9 | 0.9 | 1 | 0.02* |
| CB | | 0.03* | 0.02* | 0.23 | 0.20 | 0.9 | 0.9 | 0.01* | 0.04* | 0.02* | 1 |

Data are p-values from the post-hoc analysis. * indicates a p-value < 0.05.

ACA = anterior cerebral artery, MCA = middle cerebral artery, PCA = posterior cerebral artery, CB = cerebellum, P = proximal, M = middle, D = distal, ANOVA = analysis of variance

Supplemental Table 2. Post-hoc analysis of one-way ANOVA for the comparison of frequencies of brain metastases from adenocarcinoma according to subregions of the distal anterior cerebral artery and middle cerebral artery territories

|  | SFG | MFG | PreG | STG | PostG | SPL | ASG | PSG | AG | LOC | SMA |
| --- | --- | --- | --- | --- | --- | --- | --- | --- | --- | --- | --- |
| SFG | 1 | 0.9 | 0.55 | 0.55 | 0.9 | 0.9 | 0.13 | 0.18 | 0.13 | 0.9 | 0.10 |
| MFG | 0.9 | 1 | 0.9 | 0.10 | 0.9 | 0.49 | 0.01* | 0.01* | 0.01* | 0.9 | <0.01* |
| PreG | 0.55 | 0.9 | 1 | <0.01* | 0.9 | 0.02* | <0.01* | <0.01* | <0.01* | 0.9 | <0.01* |
| STG | 0.55 | 0.10 | <0.01* | 1 | 0.05 | 0.9 | 0.9 | 0.9 | 0.9 | 0.18 | 0.9 |
| PostG | 0.9 | 0.9 | 0.9 | 0.05 | 1 | 0.34 | <0.01* | <0.01* | <0.01* | 0.9 | <0.01* |
| SPL | 0.9 | 0.49 | 0.02* | 0.9 | 0.34 | 1 | 0.9 | 0.9 | 0.9 | 0.62 | 0.88 |
| ASG | 0.13 | 0.01* | <0.01* | 0.9 | <0.01* | 0.9 | 1 | 0.9 | 0.9 | 0.02* | 0.9 |
| PSG | 0.18 | 0.01* | <0.01* | 0.9 | <0.01* | 0.9 | 0.9 | 1 | 0.9 | 0.03* | 0.9 |
| AG | 0.13 | 0.01* | <0.01* | 0.9 | <0.01* | 0.9 | 0.9 | 0.9 | 1 | 0.02* | 0.9 |
| LOC | 0.9 | 0.9 | 0.9 | 0.18 | 0.9 | 0.62 | 0.02* | 0.03* | 0.02* | 1 | 0.01* |
| SMA | 0.10 | <0.01* | <0.01* | 0.9 | <0.01* | 0.88 | 0.9 | 0.9 | 0.9 | 0.01* | 1 |

Data are p-values from the post-hoc analysis. * indicates a p-value < 0.05.

SFG = superior frontal gyrus, MFG= middle frontal gyrus, PreG = precentral gyrus, STG = superior temporal gyrus, PostG = postcentral gyrus, SPL = superior parietal lobule, ASG = anterior supramarginal gyrus, PSG = posterior supramarginal gyrus, AG = angular gyrus, LOC = lateral occipital cortex, SMA = supplementary motor area, CB = cerebellum, LB = lobules, ANOVA = analysis of variance

Supplemental Table 3. Post-hoc analysis of one-way ANOVA for the comparison of frequencies of brain metastases from adenocarcinoma according to subregions of the cerebellum

|  | Lobules I-IV | Lobules V | Lobules VI | Crus I | Crus II | Lobules VIIb | Lobules VIIIa | Lobules VIIIb | Lobules IX | Lobules X |
| --- | --- | --- | --- | --- | --- | --- | --- | --- | --- | --- |
| Lobules I-IV | 1 | 0.9 | <0.01* | <0.01* | 0.11 | 0.9 | 0.9 | 0.9 | 0.9 | 0.9 |
| Lobules V | 0.9 | 1 | 0.02* | <0.01* | 0.36 | 0.9 | 0.9 | 0.9 | 0.9 | 0.9 |
| Lobules VI | <0.01* | 0.02* | 1 | 0.9 | 0.9 | 0.11 | 0.05 | <0.01* | <0.01* | <0.01* |
| Crus I | <0.01* | <0.01* | 0.9 | 1 | 0.77 | 0.01* | <0.01* | <0.01* | <0.01* | <0.01* |
| Crus II | 0.11 | 0.36 | 0.9 | 0.77 | 1 | 0.69 | 0.53 | 0.05 | 0.03* | 0.02* |
| Lobules VIIb | 0.9 | 0.9 | 0.11 | 0.01* | 0.69 | 1 | 0.9 | 0.9 | 0.9 | 0.85 |
| Lobules VIIIa | 0.9 | 0.9 | 0.05 | <0.01* | 0.53 | 0.9 | 1 | 0.9 | 0.9 | 0.9 |
| Lobules VIIIb | 0.9 | 0.9 | <0.01* | <0.01* | 0.05 | 0.9 | 0.9 | 1 | 0.9 | 0.9 |
| Lobules IX | 0.9 | 0.9 | <0.01* | <0.01* | 0.03* | 0.9 | 0.9 | 0.9 | 1 | 0.9 |
| Lobules X | 0.9 | 0.9 | <0.01* | <0.01* | 0.02* | 0.85 | 0.9 | 0.9 | 0.9 | 1 |

Data are p-values of the post-hoc analysis. * indicates a p-value < 0.05. ANOVA = analysis of variance

Supplemental Table 5. Post-hoc analysis of one-way ANOVA for the comparison of frequencies of brain metastases according to arterial territories when the number of brain metastases was <10

|  |  | ACA |  | ACA | MCA | MCA | MCA | PCA | PCA | PCA | CB |
| --- | --- | --- | --- | --- | --- | --- | --- | --- | --- | --- | --- |
|  |  | P | M | D | P | M | D | P | M | D |  |
| ACA | P | 1 | 1 | 1 | 1 | 1 | 1 | 1 | 1 | 1 | 1 |
| ACA | M | 1 | 1 | 1 | 1 | 1 | 0.04* | 1 | 1 | 1 | 1 |
| ACA | D | 1 | 1 | 1 | 1 | 1 | 1 | 1 | 1 | 1 | 1 |
| MCA | P | 1 | 1 | 1 | 1 | 1 | 1 | 1 | 1 | 1 | 1 |
| MCA | M | 1 | 1 | 1 | 1 | 1 | 1 | 0.01* | 0.03* | 1 | 1 |
| MCA | D | 1 | 0.04* | 1 | 1 | 1 | 1 | 0.01* | 0.02* | 0.04* | 1 |
| PCA | P | 1 | 1 | 1 | 1 | 0.01* | 0.01* | 1 | 1 | 1 | <0.01* |
| PCA | M | 1 | 1 | 1 | 1 | 0.03* | 0.02* | 1 | 1 | 1 | 0.01* |
| PCA | D | 1 | 1 | 1 | 1 | 1 | 0.04* | 1 | 1 | 1 | 1 |
| CB | | 1 | 1 | 1 | 1 | 1 | 1 | <0.01* | 0.01* | 1 | 1 |

Data are p-values from the post-hoc analysis. * indicates a p-value < 0.05.

ACA = anterior cerebral artery, MCA = middle cerebral artery, PCA = posterior cerebral artery, CB = cerebellum, P = proximal, M = middle, D = distal, ANOVA = analysis of variance

Supplemental Table 6. Post-hoc analysis of one-way ANOVA for the comparison of frequencies of brain metastases according to arterial territories when the number of brain metastases was >10

|  |  | ACA |  | ACA | MCA | MCA | MCA | PCA | PCA | PCA | CB |
| --- | --- | --- | --- | --- | --- | --- | --- | --- | --- | --- | --- |
|  |  | P | M | D | P | M | D | P | M | D |  |
| ACA | P | 1 | 0.9 | 0.9 | 0.9 | 0.02* | <0.01* | 0.9 | 0.9 | 0.9 | 0.02* |
| ACA | M | 0.9 | 1 | 0.9 | 0.9 | 0.05 | <0.01* | 0.9 | 0.9 | 0.9 | 0.04* |
| ACA | D | 0.9 | 0.9 | 1 | 0.9 | 0.45 | 0.04* | 0.9 | 0.9 | 0.9 | 0.39 |
| MCA | P | 0.9 | 0.9 | 0.9 | 1 | 0.50 | 0.05 | 0.9 | 0.9 | 0.9 | 0.45 |
| MCA | M | 0.02* | 0.05 | 0.45 | 0.50 | 1 | 0.9 | 0.04* | 0.14 | 0.04 | 0.9 |
| MCA | D | <0.01* | <0.01* | 0.04* | 0.05 | 0.9 | 1 | <0.01* | <0.01* | <0.01* | 0.9 |
| PCA | P | 0.9 | 0.9 | 0.9 | 0.9 | 0.04* | <0.01* | 1 | 0.9 | 0.9 | 0.03* |
| PCA | M | 0.9 | 0.9 | 0.9 | 0.9 | 0.14 | <0.01* | 0.9 | 1 | 0.9 | 0.11 |
| PCA | D | 0.9 | 0.9 | 0.9 | 0.9 | 0.04* | <0.01* | 0.9 | 0.9 | 1 | 0.03* |
| CB | | 0.02* | 0.04* | 0.39 | 0.45 | 0.9 | 0.9 | 0.03* | 0.11 | 0.03* | 1 |

Data are p-values from the post-hoc analysis. * indicates a p-value < 0.05.

ACA = anterior cerebral artery, MCA = middle cerebral artery, PCA = posterior cerebral artery, CB = cerebellum, P = proximal, M = middle, D = distal, ANOVA = analysis of variance
